# Pro-oxidant activity of dietary chemopreventive agents: an under-appreciated anti-cancer property

**DOI:** 10.12688/f1000research.2-135.v1

**Published:** 2013-06-04

**Authors:** Asfar S Azmi, Fazlul H Sarkar, SM Hadi

**Affiliations:** 1 Department of Pathology, Wayne State University School of Medicine, Detroit MI, 48201, USA; 2 Department of Pathology and Oncology, Karmanos Cancer Institute, Wayne State University School of Medicine, Detroit MI, 48201, USA; 3 Department of Biochemistry, Aligarh Muslim University, Aligarh, 202002, India

## Abstract

“ *Let food be thy medicine and medicine be thy food*” was quoted by Hippocrates more than two thousand years ago and since ancient times the health benefits of different natural agents have been exploited. In modern research, the disease preventive benefits of many such natural agents, particularly dietary compounds and their derivatives, has been attributed to their well recognized activity as the regulators of redox state of the cell. Nevertheless, most of these studies have focused on their antioxidant activity. A large body of evidence indicates that a major fraction of these agents can elicit pro-oxidant (radical generating) behavior which has been linked to their anti-cancer effects. This editorial provides an overview of the under-appreciated pro-oxidant activity of natural products, with a special focus on their ability to generate reactive oxygen species in the presence of transition metal ions, and discusses their possible use as cancer chemotherapeutic agents.

## Introduction

Plant derived agents (fruit and vegetable derived agents and their derivatives) have been recognized to provide a number of disease preventive properties ^1^. It is now more than 20 years since the anti-cancer properties of some of the major natural agents such as resveratrol, tea catechins (particularly epigallocatechin (EGCG)) and the turmeric compound curcumin were recognized ^2^. Their anti-cancer activity has been primarily attributed to their antioxidant behavior and the popular notion of these agents as antioxidants has driven increased consumption in developed countries ^3^. Further work in independent laboratories has shown that most of these agents can induce cell death through apoptosis, suppress anti-apoptotic pathways, block cell proliferation and modulate a number of important proteins implicated in sustaining the growth of cancer cells ^4^. Nevertheless, there is no single unifying mechanism supporting their anti-cancer effects *in vitro* and *in vivo*. Recently, researchers have utilized systems and computational techniques to pinpoint the exact pathways modulated by some of these agents ^5^. The results point to a highly promiscuous or having multiple mechanisms of action that involves modulation of multiple signaling in cancer cells ^6^. Most importantly, researchers are yet to identify the main mechanism underlying the preferential cancer selectivity elicited by these agents, which spare normal cells. These are some of the major reasons for the skepticism surrounding their health benefits, which has become a hurdle in their clinical application. In order to solidify their clinical utility, researchers have to convincingly chalk out the primary mechanism of these promising agents.

## Pro-oxidant activity of natural agents: an underappreciated activity

While the antioxidant action of dietary plant derived agents is unquestionable, over the last decade a parallel field has evolved that has convincingly presented evidence in support of their pro-oxidant (oxygen radical generating) behavior ^7^, which may be context dependent. Supporting the significance of pro-oxidant drug action, Jim Watson recently presented a strong case that most of the anti-cancer regimens (ionizing radiation, chemotherapeutics and targeted therapeutics) work either directly or indirectly by generating reactive oxygen species (ROS) ^8^.

This concept is not new. For example a PubMed search on May 21, 2013 using the keywords ‘ *natural anti-cancer agents*’ and ‘ *prooxidants*’ returns 1159 publications, while the keywords ‘ *prooxidant*’ and ‘ *anticancer agents*’ retrieve more than 11,200 papers. Various plant extract and agents such as resveratrol, catechins, EGCG, quercetin, gossypol, curcumin and caffeic acid have routinely been shown to induce damage to isolated plasmid DNA, albeit under certain conditions (e.g. in the presence of transition metal ions, especially copper ^9^). Copper is an essential metal found in chromatin, and observed to be elevated in a number of malignancies ^10^. In the late 1980s, our group was the first to describe quercetin-induced DNA strand scission in the presence of copper ^11^. Following our line of research, Fukuhara and Miyata were the first to demonstrate that resveratrol can induce oxidative DNA damage in the presence of copper ions ^12^. Such activity is dependent on a Fenton type reaction in which resveratrol oxidation results in the generation of superoxide radicals that re-oxidize to produce hydroxyl radicals in the presence of Cu(II) (elaborated in Zheng’s article ^13^). Our laboratory showed similar behavior for a number of natural agents, which led us to propose the hypothesis that one of the major anti-cancer mechanisms of plant polyphenolic compounds involves their pro-oxidant behavior in the presence of transition metal ions ^14^. Other groups have also shown similar oxidant behavior in the presence of iron ^15^.

Using an alkaline gel single cell electrophoresis/Comet assay, our group was among the first to demonstrate such pro-oxidant behavior in a cellular/biological system either alone or in the presence of copper ^16^. Since then a number of laboratories have confirmed these results ^17, 18^, which resulted in the increasing acceptance of the pro-oxidant behavior of a number of natural agents. Recently, we also showed the pro-oxidant DNA damaging effects of resveratrol in a mice model with supra-copper concentrations ^19^. These and other studies ^20^ unequivocally prove that, in addition to their antioxidant behavior, natural dietary and diet-derived agents can elicit pro-oxidant behavior and this property cannot be ignored if drawing up a design for a clinical trial investigating the anti-cancer activity of natural agents.

It has been recognized that additional work is needed to further elucidate the mechanism supporting such pro-oxidant behavior of natural agents and the reasons for their cancer cell selectivity. Key questions remain such as:

(a) Is pro-oxidant action the primary mechanism of these agents since ROS generation is a very rapid event that occurs within nano-seconds?

(b) What are the metabolic differences in tumors that switch the plant derived agents’ antioxidant behavior to pro-oxidant?

(c) Apart from DNA damage, what is the effect of such pro-oxidant behavior on other cellular components since it is well known that proteins and lipids are also prone to oxidative damage?

(d) Is there any preferential selectivity of natural agents towards copper to generate reactive oxygen species over other metal ions found in the cellular milieu?

(e) Are supra concentrations of copper or other transitional metals required to achieve such pro-oxidant behavior and does this has any value in actual patients?

Some of the answers came from very recent studies of ours in which we showed that agents such as resveratrol can take advantage of the metabolic differences in solid tumors leading to selective cancer cell killing ^21^. Our findings have demonstrated that in solid tumors, the preferential dependence of cancer cells on glycolysis (Warburg effect) leads to a lowering of tumor cell pH, which loosens the DNA structure thereby leading to exposure of chromatin-bound copper ^22^. Such labile copper is more prone to attack by resveratrol, leading to oxidative DNA damage. In non-cancerous cells, the primary source of ATP generation is through the citric acid cycle, and therefore these cells maintain physiological pH and so have normal DNA integrity. This observation is just one small step in explaining the cancer cell selectivity of natural pro-oxidant agents.

## The cell culture artifact conundrum

As convincing as it sounds, the pro-oxidant activities of natural agents come with caveats. For example, Barry Halliwell and colleagues have suggested that the pro-oxidant behavior is nothing but an artifact of cell culture conditions ^23^. They propose that most natural agents get oxidized in the presence of the cell culture media component phenol red, which can generate oxygen radicals leading to lipid peroxidation. However, these assumptions were proved to be wrong when different dietary agents were tested in phenol-free media and their pro-oxidant behavior was retained ^24^. Such experiment put to rest the controversy involving ROS generation in experimental conditions. In another study, Burkitt and colleagues showed that under normal physiological conditions, i.e. in the presence of either ascorbic acid or glutathione, resveratrol loses its pro-oxidant property and behaves as an antioxidant ^25^. In the ascorbate system, resveratrol had no effect on the rate of hydroxyl radical formation, but protected DNA from damage by acting as a radical-scavenging antioxidant. Through these studies the authors have concluded that the DNA-damaging properties of resveratrol, as identified by Fukuhara and Miyata, will be of no significance under standard physiological conditions. Nevertheless, as described above, we have shown both in cellular and animal models that their pro-oxidant behavior in tumors remains relevant ^15, 16^.

## Conclusion and future direction

As much as the anti-cancer activity of natural agents has intrigued researchers, it has also generated a lot of skepticism and controversy. Even though their promiscuous behavior is gaining some acceptance, the lack of a unifying mechanism supporting their activity has kept them at the periphery of clinical application. These agents are multi-mechanistic in nature and merely focusing on their antioxidant behavior will not do justice to their multifarious health benefits. Similarly, honing in on their effect on only a specific set of cancer hallmarks will not allow a proper understanding of the anti-cancer activity of these agents. Researchers need to pay more attention to the dogma-challenging yet under-appreciated pro-oxidant behavior of dietary agents. This exercise is expected to highlight unanswered questions and may lead to the definition of a single unifying mechanism of action related to their unquestionable anti-cancer effects. This in turn will help in the rapid clinical application of agents or their derivatives from nature’s bounty for the treatment of human malignancies.

## References

[1] Willett WC Stampfer MJ: Current evidence on healthy eating. *Annu Rev Public Health.* 2013;34:77–95 10.1146/annurev-publhealth-031811-124646 23297654

[2] Katiyar SK Agarwal R Wang ZY: (-)-Epigallocatechin-3-gallate in Camellia sinensis leaves from Himalayan region of Sikkim: inhibitory effects against biochemical events and tumor initiation in Sencar mouse skin. *Nutr Cancer.* 1992;18(1):73–83 10.1080/01635589209514207 1408948

[3] Skrovankova S Misurcova L Machu L: Antioxidant activity and protecting health effects of common medicinal plants. *Adv Food Nutr Res.* 2012;67:75–139 10.1016/B978-0-12-394598-3.00003-4 23034115

[4] Raffoul JJ Kucuk O Sarkar FH: Dietary agents in cancer chemoprevention and treatment. *J Oncol.* 2012;2012:749310 10.1155/2012/749310 23316231 PMC3536068

[5] Tan Y Wu Q Xia J: Systems biology approaches in identifying the targets of natural compounds for cancer therapy. *Curr Drug Discov Technol.* 2013;10(2):139–46 10.2174/1570163811310020006 23237676

[6] Azmi AS Mohammad RM Sarkar FH: Can network pharmacology rescue neutraceutical cancer research? *Drug Discov Today.* 2012;17(15–16):807–809 10.1016/j.drudis.2012.06.008 22727577

[7] Ullah MF Ahmad A Khan HY: The prooxidant action of dietary antioxidants leading to cellular DNA breakage and anticancer effects: Implications for chemotherapeutic action against cancer. *Cell Biochem Biophys.* 2011 10.1007/s12013-011-9303-4 22038302

[8] Watson J: Oxidants, antioxidants and the current incurability of metastatic cancers. *Open Biol.* 2013;3(1):120144 10.1098/rsob.120144 23303309 PMC3603456

[9] Hadi SM Bhat SH Azmi AS: Oxidative breakage of cellular DNA by plant polyphenols: a putative mechanism for anticancer properties. *Semin Cancer Biol.* 2007;17(5):370–376 10.1016/j.semcancer.2007.04.002 17572102

[10] Linder MC: The relationship of copper to DNA damage and damage prevention in humans. *Mutat Res.* 2012;733(1–2):83–91 10.1016/j.mrfmmm.2012.03.010 23463874

[11] Rahman A Shahabuddin Hadi SM: Strand scission in DNA induced by quercetin and Cu(II): role of Cu(I) and oxygen free radicals. *Carcinogenesis.* 1989;10(10):1833–1839 10.1093/carcin/10.10.1833 2791202

[12] Fukuhara K Miyata N: Resveratrol as a new type of DNA-cleaving agent. *Bioorg Med Chem Lett.* 1998;8(22):3187–3192 10.1016/S0960-894X(98)00585-X 9873700

[13] Zheng LF Wei QY Cai YJ: DNA damage induced by resveratrol and its synthetic analogues in the presence of Cu (II) ions: mechanism and structure-activity relationship. *Free Radic Biol Med.* 2006;41(12):1807–1816 10.1016/j.freeradbiomed.2006.09.007 17157183

[14] Hadi SM Asad SF Singh S: Putative mechanism for anticancer and apoptosis-inducing properties of plant-derived polyphenolic compounds. *IUBMB Life.* 2000;50(3):167–171 10.1080/152165400300001471 11142343

[15] Tsai YC Wang YH Liou CC: Induction of oxidative DNA damage by flavonoids of propolis: its mechanism and implication about antioxidant capacity. *Chem Res Toxicol.* 2012;25(1):191–196 10.1021/tx200418k 22148389

[16] Azmi AS Bhat SH Hanif S: Plant polyphenols mobilize endogenous copper in human peripheral lymphocytes leading to oxidative DNA breakage: a putative mechanism for anticancer properties. *FEBS Lett.* 2006;580(2):533–538 10.1016/j.febslet.2005.12.059 16412432

[17] Tyagi A Gu M Takahata T: Resveratrol selectively induces DNA Damage, independent of Smad4 expression, in its efficacy against human head and neck squamous cell carcinoma. *Clin Cancer Res.* 2011;17(16):5402–5411 10.1158/1078-0432.CCR-11-1072 21705453 PMC3156958

[18] Michels G Watjen W Weber N: Resveratrol induces apoptotic cell death in rat H4IIE hepatoma cells but necrosis in C6 glioma cells. *Toxicology.* 2006;225(2–3):173–182 10.1016/j.tox.2006.05.014 16843582

[19] Khan HY Zubair H Ullah MF: Oral administration of copper to rats leads to increased lymphocyte cellular DNA degradation by dietary polyphenols: implications for a cancer preventive mechanism. *Biometals.* 2011;24(6):1169–1178 10.1007/s10534-011-9475-9 21717118

[20] Martin-Cordero C Leon-Gonzalez AJ Calderon-Montano JM: Pro-oxidant natural products as anticancer agents. *Curr Drug Targets.* 2012;13(8):1006–1028 10.2174/138945012802009044 22594470

[21] Muqbil I Beck FW Bao B: Old wine in a new bottle: the Warburg effect and anticancer mechanisms of resveratrol. *Curr Pharm Des.* 2012;18(12):1645–1654 10.2174/138161212799958567 22288443

[22] Shamim U Hanif S Albanyan A: Resveratrol-induced apoptosis is enhanced in low pH environments associated with cancer. *J Cell Physiol.* 2012;227(4):1493–1500 10.1002/jcp.22865 21678400 PMC3185178

[23] Long LH Clement MV Halliwell B: Artifacts in cell culture: rapid generation of hydrogen peroxide on addition of (-)-epigallocatechin, (-)-epigallocatechin gallate, (+)-catechin, and quercetin to commonly used cell culture media. *Biochem Biophys Res Commun.* 2000;273(1):50–53 10.1006/bbrc.2000.2895 10873562

[24] Sudheer AR Muthukumaran S Devipriya N: Ellagic acid, a natural polyphenol protects rat peripheral blood lymphocytes against nicotine-induced cellular and DNA damage *in vitro*: with the comparison of N-acetylcysteine. *Toxicology.* 2007;230(1):11–21 10.1016/j.tox.2006.10.010 17188416

[25] Burkitt MJ Duncan J: Effects of trans-resveratrol on copper-dependent hydroxyl-radical formation and DNA damage: evidence for hydroxyl-radical scavenging and a novel, glutathione-sparing mechanism of action. *Arch Biochem Biophys.* 2000;381(2):253–263 10.1006/abbi.2000.1973 11032413

