# Supplementary material for: Pro-oxidant activity of dietary chemopreventive agents: an under-appreciated anti-cancer property
Source: F1000Res. 2013 Jun 4;2:135. [Version 1] doi: 10.12688/f1000research.2-135.v1 (PMC3790610; doi:10.12688/f1000research.2-135.v1)
Supplement: Peer review history

# Peer review: Referee response for version 1

**Published:** 2013-09-04

**Authors:** Surinder Batra

**Affiliations:** 1 University of Nebraska, Lincoln NE, USA

This is well-written manuscript.

I have read this submission. I believe that I have an appropriate level of expertise to confirm that it is of an acceptable scientific standard.

# Peer review: Referee response for version 1

**Published:** 2013-06-05

**Authors:** Hasan Mukhtar

**Affiliations:** 1 Department of Dermatology, School of Medicine and Public Health, University of Wisconsin, Madison, WI, USA

Very well written. Was a joy to read.

I have read this submission. I believe that I have an appropriate level of expertise to confirm that it is of an acceptable scientific standard.
